# Un cas de kyste ovarien géant au cours de la grossesse

**DOI:** 10.11604/pamj.2024.47.182.43149

**Published:** 2024-04-12

**Authors:** Imen Ben Farhat, Haifa Bergaoui

**Affiliations:** 1 Université de Monastir, Faculté de Médecine de Monastir, Monastir, Tunisie,; 2 Service de Gynécologie Obstétrique du Centre de Maternité et de Néonatologie de Monastir, Monastir, Tunisie

**Keywords:** Grossesse, masse annexielle, kyste ovarien, échographie, Pregnancy, adnexal mass, ovarian cyst, ultrasound

## Abstract

The incidence of ovarian cysts in pregnancy has risen considerably since ultrasound was first routinely performed in pregnant women. This incidence is between 0.3% and 5.4%. The main risk of complication of ovarian cysts during pregnancy is adnexal torsion, estimated at around 8%. Ultrasound remains the gold standard for identifying ovarian tumors during pregnancy. A part from complications, expectant management is recommended during pregnancy in view of the maternal-fetal risk of surgery. When surgery is indicated, laparoscopy is the preferred approach during the 1^st^ and 2^nd^ trimesters. The ovarian tumor should not affect the delivery procedure, except in the case of a complication of suspected malignancy or a previa obstruction, as in the case of our patient. We present a 30-year-old patient with no pathological medical history who underwent surgery for a benign ovarian cyst 10 years ago, G3P3, unscarred uterus, who consulted us at 38 SA for pregnancy monitoring. In addition, there was only one prenatal consultation during the follow-up, with an ovarian cyst measuring 5 cm at the first trimester ultrasound. During the obstetric ultrasound, we noted the presence of a 13 cm unilocular anechogenic cystic image with a regular wall, creating a Previa obstruction. A prophylactic caesarean section was performed with simultaneous cystectomy without incident.

## Image en médecine

L´incidence des kystes ovariens au cours de la grossesse a considérablement augmenté depuis la pratique systématique de l´échographie chez la femme enceinte. Cette fréquence est comprise entre 0,3 et 5,4%. Le principal risque de complication des kystes ovariens durant la grossesse est la torsion annexielle, évaluée autour de 8%. L´échographie reste l´examen de référence pour caractériser une tumeur ovarienne durant la grossesse. En dehors des complications, l´expectative est recommandée au cours de la grossesse vue le risque maternofoetal de la chirurgie. Lorsqu´un acte chirurgical est indiqué, la cœlioscopie est la voie d´abord de choix au cours du 1^er^ et du 2^e^ trimestres. Les modalités de l´accouchement ne doivent pas être modifiées par la tumeur ovarienne, excepté en cas de complication de suspicion de malignité ou d´obstacle prævia comme le cas de notre patiente. Nous présentons le cas d´une patiente âgée de 34 ans sans antécédents médicaux pathologique opérée pour un kyste ovarien bénin il y 10 ans, G3P3, utérus sain qui nous consulte à 38 SA pour suivi de la grossesse. Par ailleurs, une seule consultation prénatale a été faite au cours de suivi avec à l´échographie de premier trimestre. Un kyste ovarien de 5cm. Nous avons relevé lors de la réalisation de l´échographie obstétricale la présence d´une image kystique uniloculaire anéchogène pure à paroi régulière faisant un obstacle prævia. Une césarienne prophylactique a été décidée avec kystectomie en même temps passées sans incidents.

**Figure 1:**
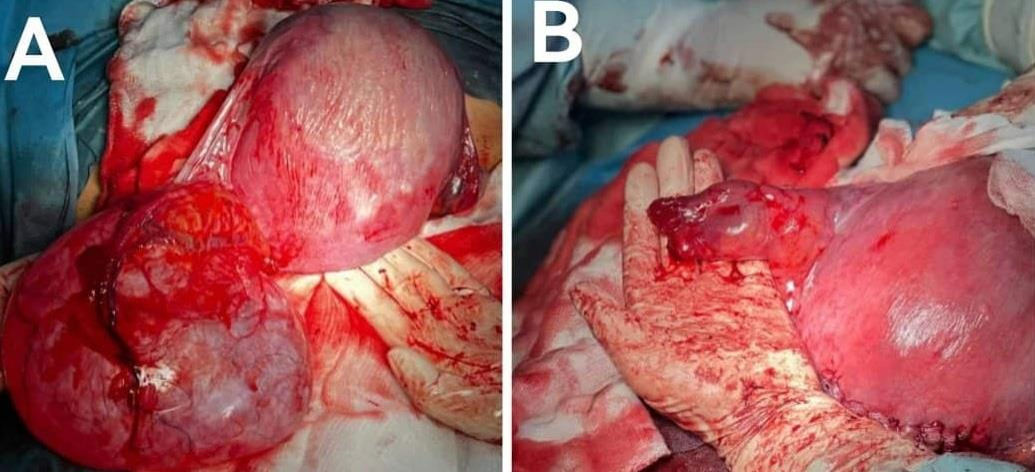
A) aspect per opératoire du kyste de l’ovaire de 13cm réalisant un obstacle prævia ; B) aspect après kystectomie

